# DNMT1 ablation suppresses tumorigenesis by inhibiting the self-renewal of esophageal cancer stem cells

**DOI:** 10.18632/oncotarget.24116

**Published:** 2018-01-02

**Authors:** Ying Teng, Xiying Yu, Hui Yuan, Liping Guo, Wei Jiang, Shih-Hsin Lu

**Affiliations:** ^1^ Department of Etiology and Carcinogenesis and State Key Laboratory of Molecular Oncology, National Cancer Center/Cancer Hospital, Chinese Academy of Medical Sciences and Peking Union Medical College, Beijing, China; ^2^ Beijing Key Laboratory for Carcinogenesis and Cancer Prevention, National Cancer Center/Cancer Hospital, Chinese Academy of Medical Sciences, Beijing, China; ^3^ Department of Oncology, The First Affiliated Hospital of Zhengzhou University, Zhengzhou, China

**Keywords:** cancer stem cells, esophageal cancer, ESCC, DNMT1, 5-aza-dC

## Abstract

Cancer stem cells (CSCs) have been isolated from many tumors and considered as the main reason of cancer recurrence and metastasis. DNA methyltransferase 1 (DNMT1) mediates DNA methylation and plays an important role in CSCs maintenance. However, the function of DNMT1 in CSCs of esophageal squamous cell carcinoma (ESCC) remains unclear. In this study, we examined the role of DNMT1 in regulating self-renewal in CSCs of ESCC. We found a high expression of DNMT1 in both side population (SP) cells and sphere formation cells that represented as substitutes for CSCs in KYSE150 and EC109 ESCC cell lines. We performed the knockdown of DNMT1 using lentivirus-mediated RNA interference (RNAi) methods. We revealed that ablation of DNMT1 resulted in the numbers and self-renewal abilities of CSCs refrained significantly in ESCC cells. As a result of the CSCs inhibition, the malignant phenotypes such as cell proliferation, colony formation, migration and drug resistance abilities were dramatically inhibited in ESCC cells. Treatment of 5-aza-2'-deoxycytidine (5-aza-dC), a DNMT inhibitor, also resulted in the inhibition of CSCs and malignant profiles in ESCC cells. Our findings also provided the first evidence that 5-aza-dC inhibited the colony and sphere formation of CSCs. Thus, our results indicated that DNMT1 was important for the self-renewal maintenance of CSCs in ESCC, and 5-aza-dC could be a potential therapy for the CSCs of ESCC.

## INTRODUCTION

Esophageal cancer is a common cancer in the world [1]. In China, esophageal squamous cell carcinoma (ESCC) is the most common histological subtype, accounts for almost 90% of all esophageal cancers, and is the 3rd leading cause of cancer morbidities and 4th cancer mortalities [2, 3]. The recurrence and metastasis rates of ESCC are very high and the prognosis is usually poor [4]. The main reason is likely due to the residual malignant cells in tumor with stem-cell-like potential. These tiny amount cells are called as cancer stem cells.

The cancer stem cells (CSCs) play pivotal role in carcinogenesis, progression and metastasis. CSCs possess higher resistance to chemotherapy/radiotherapy than cancer cells, which can result in ineffective treatment and cancer relapse after treatment [5]. CSCs were first identified in hematological malignancies, mainly in acute myelogenous leukemia [6]. Then CSCs were found in solid tumors [7–11]. In our previous studies, we isolated CSCs of ESCC using side population (SP) sorting with Hoechst 33342, and our results showed that SP cells shared certain common features of cancer stem cells, such as the ability of self-renewal, highly proliferative and tumorigenic, and expressing several stem cell-related genes, including SOX2, OCT-4, BMI-1 and so on [12, 13]. We also used sphere-forming assay to isolate CSCs in ESCC cells, and found that these cells overexpress stem cell-related genes. Based on our previous research, we demonstrated that SP cells and sphere formation cells could be good models for CSCs in ESCC cell lines.

DNA methyltransferase 1(DNMT1) is a member of the DNA methyltransferase family and mediates DNA methylation. DNMT1 is responsible for maintaining methylation patterns of stem cells [14–16]. Recent evidence demonstrated that DNMT1 played an important role in the maintenance of CSCs [17–20]. The functions of DNMT1 in the regulation of esophageal cancer stem cells have not been studied.

In this study, we investigated the role of DNMT1 in esophageal cancer stem cells. Our results demonstrated that DNMT1 played important roles in the self-renewal maintenance of ESCC-CSCs, and 5-aza-dC, a DNMT inhibitor, could be a potential therapy for the CSCs of ESCC.

## RESULTS

### The expression of DNMT1 is higher in esophageal CSCs

**Figure 1:**
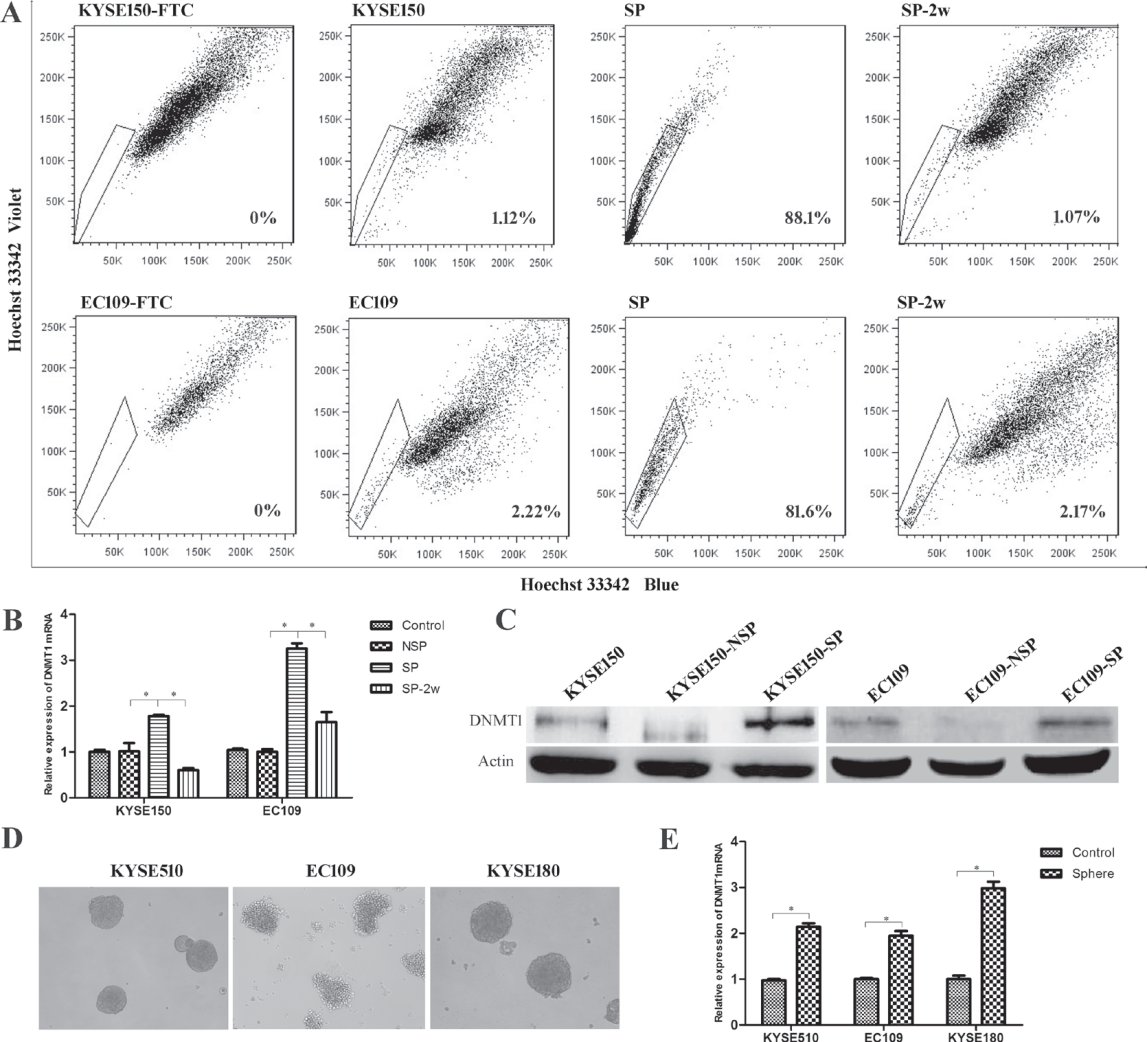
The expression of DNMT1 in esophageal cancer stem cells (**A**) Side population (SP) analysis of KYSE150 and EC109 cell lines. (**B**) The mRNA of DNMT1 in SP cells, and the expression was decreased during the differentiation from SP to NSP cells. *P* < 0.05. (**C**) Western bolt detected an increased protein expression of DNMT1 in SP cells. (**D**) Tumor spheres formed by ESCC cell lines. E. The expression of DNMT1 in sphere cells is higher than the parental populations in mRNA level.

Side population (SP) cells were isolated by FACS, and represented as substitutes for CSCs in KYSE150 and EC109 ESCC cell lines. The SP cells were continually cultured for two weeks. We found that the percentage of SP cells is decreased to normal levels because of differentiation of SP cells to non-SP cells (Figure 1A). We found a high expression of DNMT1 in SP cells of KYSE150 and EC109 cells lines both in mRNA and protein level (Figure 1B and 1C). Interestingly, the mRNA expression of DNMT1 was decreased during the differentiation from SP to non-SP cells (Figure 1B). We detected the expression of DNMT1 in spheroid formation cells which is another method of enriching CSCs from KYSE510, KYSE180 and EC109 cell lines, and we found that the mRNA level of DNMT1 was higher than that of parental populations (Figure 1D–1E).

### Reduction of DNMT1 leads to low rates of CSCs

**Figure 2:**
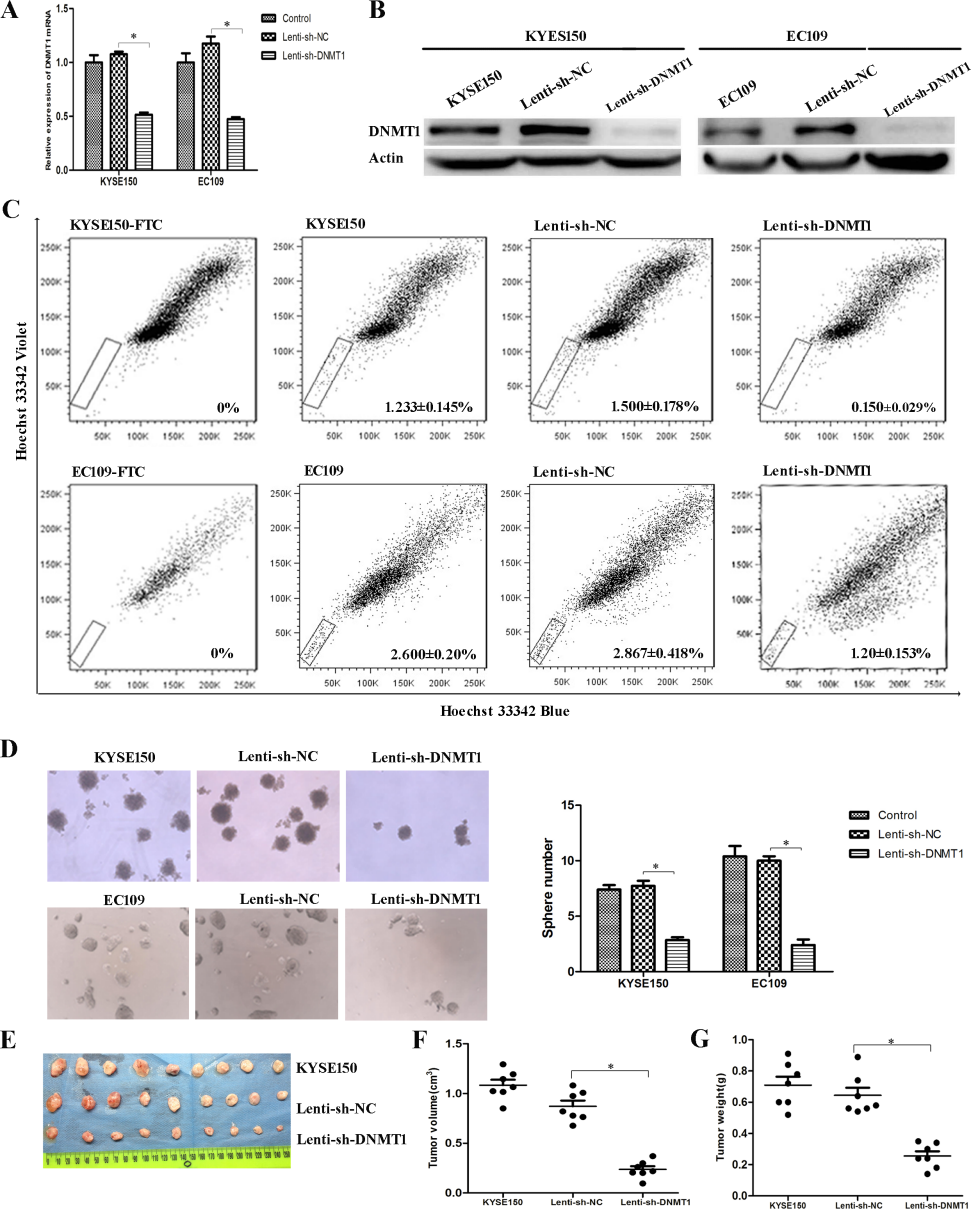
Reduction of DNMT1 led to low rates of CSCs (**A**) The expression of DNMT1 mRNA was decreased as a result of lentivirus-mediated RNA interference (RNAi). (**B**) The protein expression of DNMT1 is down-regulated in Lenti-sh-DNMT1 transfected cells. (**C**) Lenti-sh-DNMT1 transfection resulted in decreased CSCs by SP analysis. (**D**) Sphere formation abilities were inhibited in Lenti-sh-DNMT1 group. (**E**–**F**) The volumes of tumor xenografts from Lenti-sh-DNMT1 group were much smaller than those from KYSE150 and Lenti-sh-NC group. (**G**) The weights of tumor xenografts from Lenti-sh-DNMT1 group were much lighter than those two groups. *P* < 0.05.

In order to further validate the effect of DNMT1 in ESCC-CSCs, we transfected Lenti-sh-DNMT1 virus to KYSE150 and EC109 cells for knockdown the expression of DNMT1. The expression of DNMT1 was dramatically knocked down in mRNA (Figure 2A) and protein (Figure 2B) level. DNMT1 has been described to be essential for maintenance of stem cells. Therefore CSC population in KYSE150 and EC109 cells were analyzed by SP analysis (Figure 2C). In KYSE150 cells, the SP cell rate in Lenti-sh-NC group was 1.500 ± 0.178%, whereas the rate in Lenti-sh-DNMT1 transfected cells was 0.150 ± 0.029%. In EC109 cells, the SP fractions of Lenti-sh-DNMT1 group were also significantly lower than Lenti-sh-NC group (2.867 ± 0.418% vs. 1.200 ± 0.513%, *P* < 0.05). We also detected the amount of CSCs by sphere formation assays, and found that the size and number of the spheres in Lenti-sh-DNMT1 group were much smaller than that of Lenti-sh-NC group (Figure 2D). These results indicate that DNMT1 regulates the fractions and self-renewal of CSCs in ESCC cell lines.

### Decrease of DNMT1 suppresses tumorigenic ability *in vivo*

Furthermore, tumorigenic ability was evaluated *in vivo* by injecting cells into nude mice. The measurement of tumor sizes showed that the volumes of tumor xenografts from Lenti-sh-DNMT1 group were much smaller than those from Lenti-sh-NC and KYSE150 groups (Figure 2E and 2F). Similarly, weights of xenografts from Lenti-sh-DNMT1 group were much lighter than those two groups (Figure 2G).

### Decrease of DNMT1 suppresses proliferation, colony formation, migration and drug resistance abilities *in vitro*

**Figure 3:**
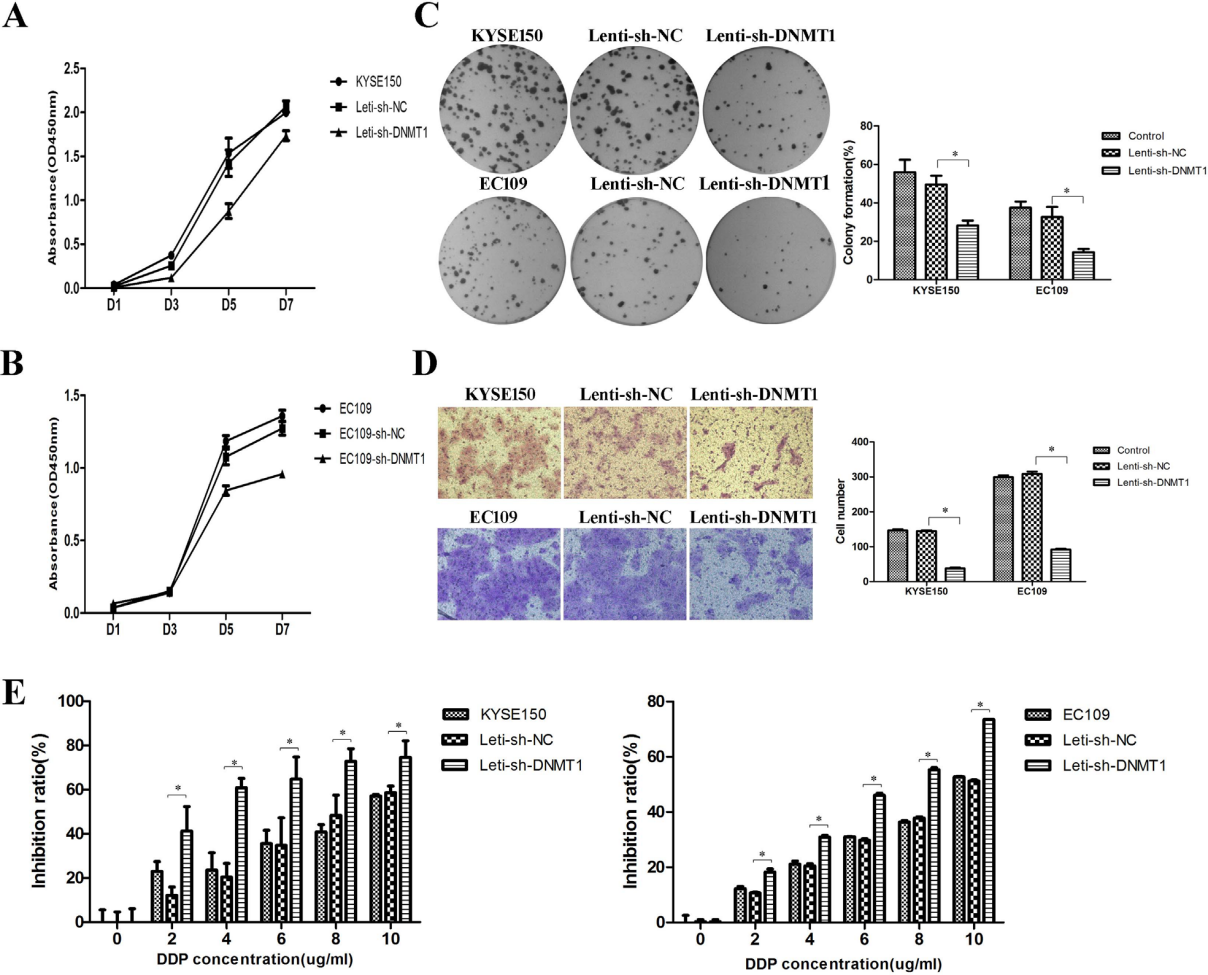
Reduction of DNMT1 suppressed proliferation, clone formation, migration and drug resistance abilities in ESCC cells (**A**–**B**) The proliferation ability was inhibited. (**C**) The clone formation ability was down-regulated. *P* < 0.05. (**D**) The migration ability was suppressed. (**E**) The drug resistance ability was also inhibited. *P* < 0.05.

To test whether the lower number of CSCs influenced the malignant properties of ESCC cells, we performed cell proliferation analysis by CCK8 firstly. We found that knockdown of DNMT1 resulted in a significant reduction of cell proliferation (Figure 3A and 3B). In colony formation assay, we observed that colony formation abilities were inhibited in Lenti-sh-DNMT1 transfected cells (Figure 3C). And the migration experiment showed reduced migration abilities in Lenti-sh-DNMT1 virus transfected cells (Figure 3D). As reported that the high expression of DNMT1 supported the potential contribution of ovarian cancer stem cells to platinum resistance [21], we performed drug sensitivity assays by CCK8 with cisplatin (DDP) which are commonly used of chemotherapy for ESCC. As shown in Figure 3E, the inhibition ratios of cells transfected with Lenti-sh-DNMT1 virus vectors were higher than Lenti-sh-NC group in all DDP concentrations. The results demonstrated the Lenti-sh-DNMT1 transfected cells became sensitive to DDP.

### 5-aza-dC inhibits the ratios and self-renewal abilities of CSCs

**Figure 4:**
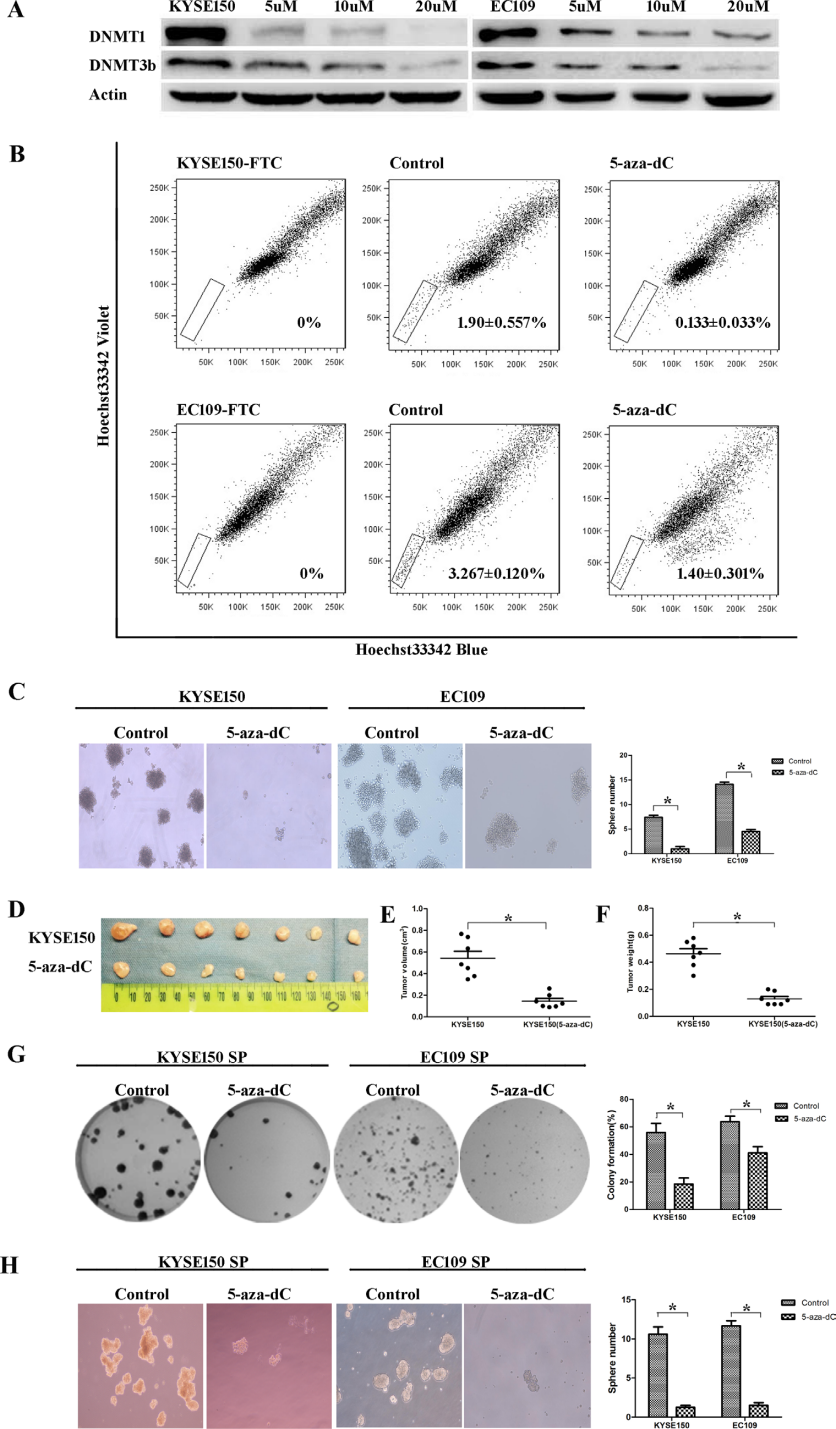
Treatment with 5-aza-dC decreasesd the number and the self-renewal abilities of ESCC-CSCs (**A**) 5-aza-dC led to the degradation of DNMT1 and DNMT3b. (**B**) 5-aza-dC treated cells showed a decreased percentage of SP cells. (**C**) The 5-aza-dC group showed decreased sphere formation ability. (**D**–**F**) Tumorigenic ability was inhibited. (**G**) Treating SP cells with 5-aza-dC suppressed their colony formation abilities. (**H**) Treating SP cells with 5-aza-dC inhibited their sphere formation capabilities.

We treated KYSE150 and EC109 cells with 5-aza-dC at different concentrations (5uM, 10uM, 20uM) for 3 days. We found that the level of DNMT1 was obviously decreased by Western blot analysis. The level of DNA methyltransferase 3b (DNMT3b) was also degraded after this treatment (Figure 4A). Then we determined CSC population by SP analysis. We found that the percentage of SP cells in the 5-aza-dC group was dramatically decreased compared with control group (Figure 4B). The numbers of tumor spheres formed by 5-aza-dC group were less than control group (Figure 4C). The volume and the weight of tumor xenografts from 5-aza-dC group were both smaller than control group *in vivo* (Figure 4D–4F). All the results reveal that 5-aza-dC could eradicate CSCs in ESCC.

To further investigated the effect of 5-aza-dC on CSCs’ self-renewal ability, colony formation and spheroid formation assays of SP cells derived from KYSE150 and EC109 cells were performed. SP cells treated with 5-aza-dC expressed lower colony formation abilities than control group (Figure 4G). Treating SP cells with 5-aza-dC also inhibited their sphere formation capabilities (Figure 4H). The results indicated that 5-aza-dC could blunt the self-renewal abilities of CSCs in ESCC.

### Inhibition of CSCs by 5-aza-dc decreases proliferation, colony formation, migration and drug resistance abilities in ESCC cells

**Figure 5:**
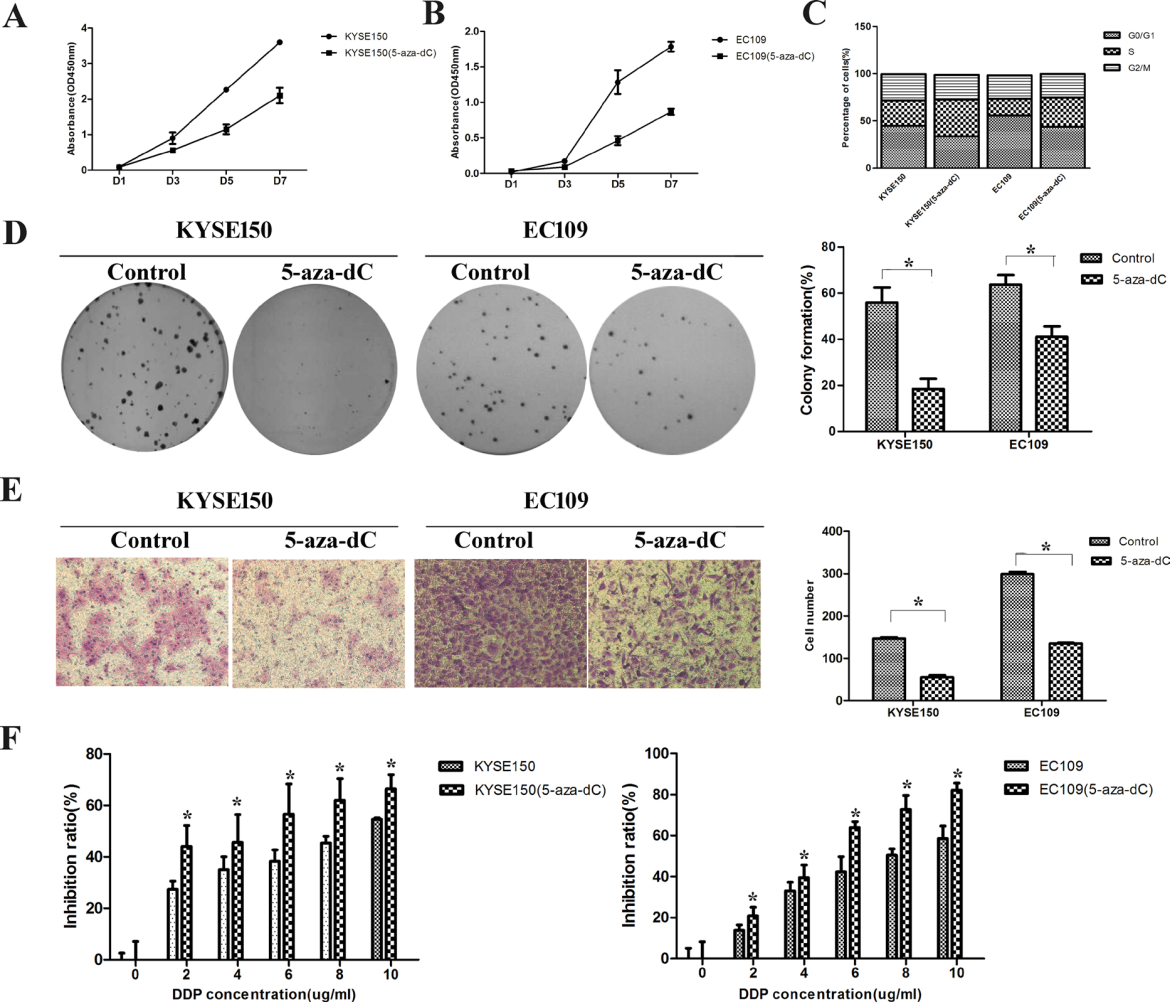
Treatment with 5-aza-dC led to downregulation of malignant properties in ESCC cells (**A**–**B**) The proliferation ability was inhibited. (**C**) The cell cycle was blocked in S phase after 5-aza-dC treatment. (**D**) The clone formation ability was down-regulated. (**E**) The migration ability after 5-aza-dC treatment was suppressed. (**F**) The drug resistance ability was inhibited.

To investigate whether the stimulation of 5-aza-dC could influence the malignant properties of ESCC cells, we performed a proliferation experiment at first. After treated with 5-aza-dC (10 uM) for 3 days, the proliferation abilities of KYSE150 and EC109 cells were inhibited (Figure 5A and 5B). Treatment with 5-aza-dC could block the cell cycle in S phase, which may be the reason of proliferation inhibition (Figure 5C). In both cell lines, colony formation and migration abilities were decreased after 5-aza-dC treatment (Figure 5D and 5E). Similarly, cells treated with 5-aza-dC showed reduced drug resistance abilities in all DDP concentrations (Figure 5F).

### DNMT1 regulates SOX2 and miRNAs expression

**Figure 6:**
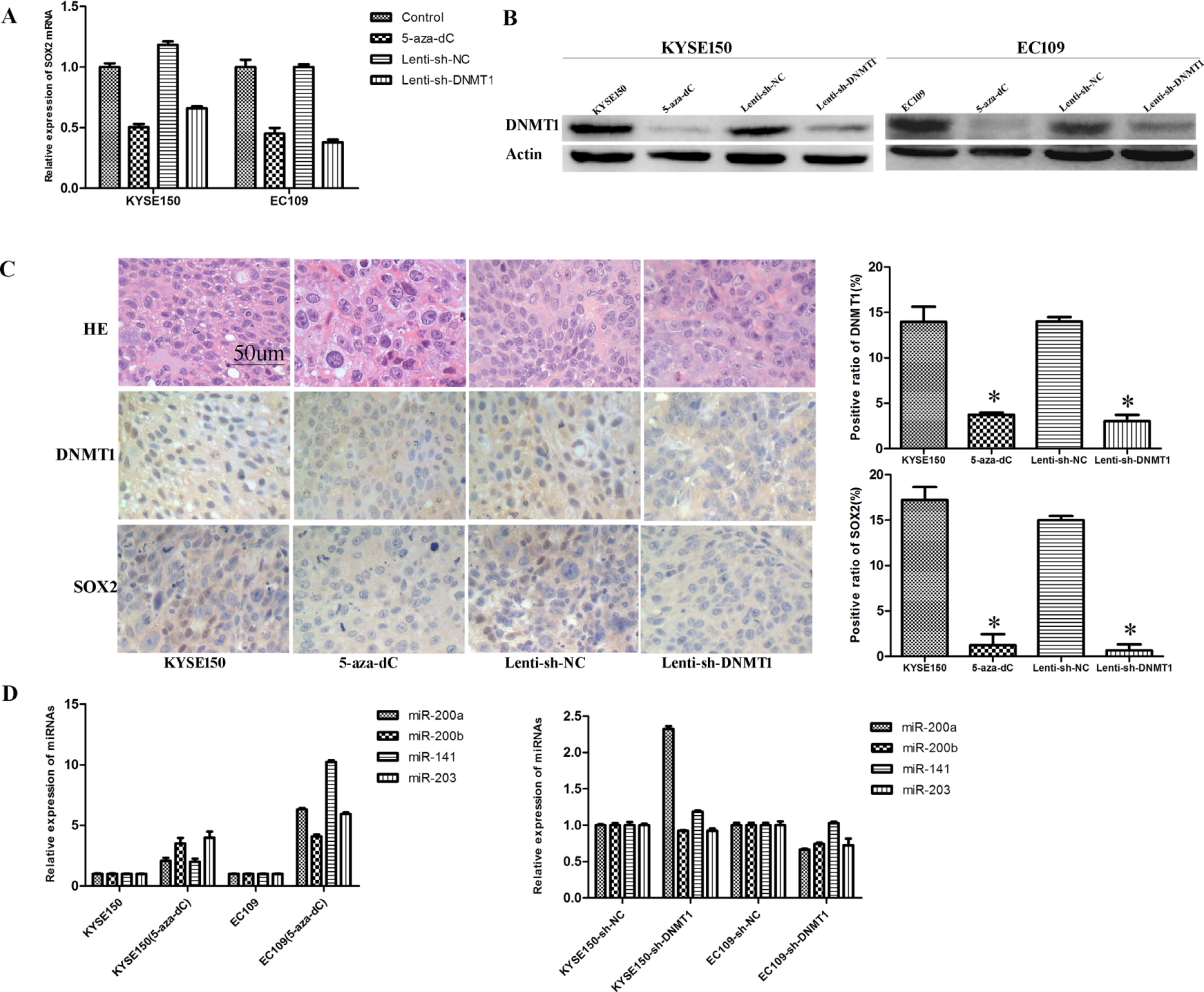
Inhibition of DNMT1 regulates the expression of SOX2 and miRNAs (**A**) The expression of SOX2 mRNA is down-regulated in DNMT1 inhibited cells. (**B**) Western bolt detected a decreased expression of SOX2. (**C**) IHC staining detected low expression of SOX2 and DNMT1in the tumors from 5-aza-dC and Lenti-sh-DNMT1 groups. (**D**) The expression of miR-203, miR-141, miR-200A and miR-200B were up-regulated after 5-aza-dC treatment.

The ablation of DNMT1 expression by Lenti-sh-DNMT1 transfection or the treatment of 5-aza-dC resulted in the decreased expression of SOX2 both in mRNA and protein level (Figure 6A and Figure 6B). Using immunohistochemistry assay we found a decreased expression of SOX2 in Lenti-sh-DNMT1 or 5-aza-dC treated tumor xenografts (Figure 6C). MiR-200 family (including miR-200A, miR-200B and miR-141) and miR-203 were reported as key regulators to suppress “stemness” in many kinds of stem cells [22–24]. So we detected the expression of these miRNAs by real-time PCR analysis. We found that all of these miRNAs were up-regulated after the treatment of 5-aza-dC. But in DNMT1 knock-down experiments, we only detected the up-regulation of miR-200A in KYSE150 cells (Figure 6D).

## DISCUSSION

Cancer is most likely a disease of stem cells. More and more studies suggest that CSCs play an important role in the formation and progression of tumor [25, 26]. Therefore, CSCs may be the ultimate target in cancer therapy.

CSCs have many stem cell properties, but the mechanisms to maintain these properties remain unclear. DNMT1 is responsible for maintaining methylation patterns. In previous studies, the over-expressions of DNMT1 have been reported in the human cancers [27, 28]. Recent studies indicate that DNMT1 is essential for maintenance of stem cells and CSCs, such as haematopoietic stem cells (HSCs)/progenitor cells [14], epidermal progenitor cells [15], mesenchymal stem cells [16] and leukemia stem cells [17]. However, no study has been mentioned the relations between DNMT1 and CSCs in ESCC. In our study, we found that DNMT1 was up-regulated in CSCs from KYSE150 and EC109 ESCC cell lines. In addition, the expression of DNMT1 was decreased during the differentiation progression of CSCs.

To further investigate the effect of DNMT1 in ESCC-CSCs, we silenced the expression of DNMT1 in KYSE150 and EC109 ESCC cells using lentivirus-mediated RNA interference (RNAi). We revealed that ablation of DNMT1 resulted in the numbers and self-renewal abilities of CSCs refrained significantly in ESCC cells. As a result of the CSCs inhibition, the malignant phenotypes such as cell proliferation, colony formation, migration and drug resistance abilities were dramatically inhibited in ESCC cells. These results are consistent with the effects of DNMT1 in CSCs reported in other cancers [18–20]. In colon cancer, suppression of DNMT1 is sufficient to exhaust CSCs and inhibit tumor-initiating ability [18]. The mammary gland-specific DNMT1 deletion protects mice from mammary tumorigenesis by limiting the CSCs pool [19]. In pancreatic ductal adenocarcinoma, CSCs expressed higher DNMT1 levels than non-CSCs, deletion of DNMT1 in CSCs reduced their self-renewal and tumorigenic potential [20]. It is also known that CSCs possess unlimited proliferation potential, ability to self-renewal and capacity to generate a progeny of differentiated cells that constitute the major tumor population. So these cells are responsible for tumor malignant phenotypes maintenance, such as chemo-radiation resistance, metastasis, and recurrence [29]. In our study, as a result of refrained CSCs by ablation of DNMT1, the malignant phenotypes of the bulk cancer cells in KYSE150 and EC109 ESCC cell lines were inhibited, including cell proliferation, colony formation, migration and drug resistance abilities.

DNA methyltransferase inhibitors, 5-aza-2’-deoxycytidine (5-aza-dC) is a strong inducer of DNA de-methylation, which act as ‘fraudulent bases’ mimicking cytosine, and once incorporated into DNA in S phase are able to trap DNMTs. Trapped DNMTs are degraded by the proteasome [30]. In our study, we found a dramatic decrease of DNMT1 and DNMT3b at protein level after the 5-aza-dC treatment. We overexpressed DNMT1 in KYSE150 cells using lentivirus-mediated transfection. Then we treated the transfected cells with 5-aza-dC. Unfortunately, after the treatment, the expression of DNMT1 was also dramatically decreased, similarly to non-transfected cells (Supplementary Figure 1).

5-aza-dC treatment led to the fraction reduction and a dramatic inhibition of self-renewal of CSCs in ESCC. This finding is consistent with previous reports that 5-aza-dC could target CSCs. Studies showed that treatment of prostate cancer stem cells with 5-aza-dC could reduce the expression of stem cell-associated genes and induced differentiation of the prostate cancer stem cells [31]. Tsai et al [32] have recently shown that the application of 5-aza-dC has successfully reduced stem cell characteristics of leukemia. Pancreatic cancer stem cells have been shown to be sensitive to 5-aza-dC, which reduces tumor sphere formation and induces apoptosis of the cancer stem cells [33]. 5-aza-dC has also been demonstrated induce differentiation of the pancreatic cancer progenitor cell lines [34, 35]. Our study also showed that treatment with 5-aza-dC could reduce proliferation, clone formation, migration and drug resistance abilities of ESCC cells. Our work shed light on development of novel clinical regimes that 5-aza-dC may be an effective therapy for ESCC patients by targeting CSCs.

Methylation is responsible for transcriptional repression in cells. Thus, it is conceivable to predict that treatment with demethylating agents would further upregulate gene expression. However, after DNMT1 inhibition or 5-aza-dC treatment, we observed a downregulation of SOX2, a stemness transcriptional regulator. The results indicate that other epigenetic regulators are likely attending the regulation of SOX2. In liver CSCs, histone deacetylatase sirtuin 1 (SIRT1) could regulates transcription of the SOX2 gene by way of chromatin-based epigenetic changes [36]. Yuan et al found that histone demethylases KDM4C, also can epigenetically enhancing SOX2 expression in tumor-initiating cell populations in human esophageal squamous cell carcinoma [37]. Our previous studies found that miRNAs played an important role in CSCs. The expression of miR-203 was down-regulated in SP cells isolated from ESCC cell lines and regulated self-renewal ability of CSCs through inhibiting Bmi-1 expression [23]. miR-141 was also down-regulated in SP of ESCC cell lines and overexpression of miR-141 could abolish the self-renewal ability and carcinogenicity of esophageal cancer stem-like cells by suppressing the expression of TM4SF1 [24]. By Reduced Representation Bisulfite Sequencing, we detected that miR-200A and miR-200B were hyper-methylated in SP cells isolated from KYSE150 cells (data was not shown here). The expressions of these four miRNAs were up-regulated after the treatment of 5-aza-dC. But in DNMT1 knock-down cells, we could not detect the up-regulation of all these miRNAs. A possible reason was 5-aza-dC could not only promote the degradation of DNMT1, but also other DNMTs such as DNMT3b which may play more important role in the methylation regulation of miRNAs. Following DNMT1 knock-down or 5-aza-dC treatment, downregulation of SOX2 and reactivation of CSC inhibitory miRNAs might partially contribute to the inhibition of ESCC-CSCs, but the exact mechanism need a further study.

In conclusion, we verified that DNMT1 play an important role in the maintenance of ESCC-CSCs. Targeting DNMT1 may improve the clinical outcome of ESCC patients as a result of CSCs eradication. 5-aza-dC, a DMNT inhibitor, may become an effective therapy for ESCC patients by targeting CSCs.

## MATERIALS AND METHODS

### Ethics statement

Investigation has been conducted in accordance with the ethical standards and according to the Declaration of Helsinki and according to national and international guidelines and has been approved by the ethics committees of Chinese Academy of Medical Sciences, Cancer hospital review board.

### Cell culture

The human ESCC cell line, KYSE150 was cultured in RPMI 1640 (BIOROC, China) supplemented with 10% fetal bovine serum (FBS) (Gibco, USA). EC109 was cultured in DMEM (BIOROC, China) supplemented with 10% fetal bovine serum (FBS) (Gibco, USA). Both cell lines were routinely incubated at 37°C in a humidified atmosphere of 5% CO_2_.

### Treatment with 5-aza-2′-deoxycytidine (5-aza-dC)

The KYSE150 and EC109 cells were treated with 5-aza-dC (Sigma-Aldrich) at different concentrations (5 uM, 10 uM, 20 uM), and finally we chose 10 uM for the next research because of obvious inhibition of DNMT1 and low toxicity. Cells were incubated with 5-aza-dC for 72 h and the culture media were replaced every 24 h with fresh media containing 5-aza-dC. Immediately following drug treatment (72 h), cells were washed twice with PBS and allowed to continue other experiments under regular drug-free conditions.

### RNA interference and transfection

Sh-RNA constructs targeting DNMT1 were cloned to lentiviral vector pSIH1-H1-Puro. Lenti-sh-NC was generated as a control. The transfection was performed according to the instruction of the Lipofectamine2000 (Invitrogen). Then, the cells were selected with a standard medium containing 2 μg/ml puromycin (Gibco) for 14 days, and puromycin-resistance colonies were pooled to establish stable transfectants. The stable transfected cells were then used for subsequent studies.

### Side population (SP) cell sorting by Fluorescence activated Cell Sorting (FACS)

SP sorting was performed as previously described [23]. KYSE150 cells were incubated with Hoechst 33342 (Sigma) at a final concentration of 5 μg/ml. The concentration for EC109 cells were 7.5 μg/ml.

### RNA isolation and Real-time quantitative RT-PCR

Total cellular RNA was extracted using the Trizol reagent (Ambion, USA) according to the manufacturer’s instruction. The relative expression levels of DNMT1 and SOX2 were carried out according to the standard protocol of SYRB Premix Ex Taq^™^ Perfect Real Time system (Takara, Dalian, China). The expression of miRNAs was measured by a two-step Taqman assay (Applied Biosystems, USA). GAPDH or U6 was used as the internal control. The primers used were as follows: DNMT1—F: CCATCAGGCATTCTACCA; DNMT1—R: CGTTCTCCTTGTCTTCTCT; SOX2—F: GCTCCTACCGTACCACTAGAACTT; SOX2—R: TCGTTCTTGTTATTACGCTGTTTT; GAPDH-F: GAGTCAACGGATTTGGTCGT; GAPDH—R: GACAAGCTTCCCGTTCTCAG. The PCR reaction was performed with a StepOne Real-time PCR Systems (Applied Biosystems, USA). The relative expression of genes was calculated using the 2^-ΔΔCt^ method.

### Western blot analysis

Harvested cultured cells were lysed in RIPA lysis buffer (PPLYGEN, C1053). The concentration of proteins in cell lysates was quantified by the BCA Protein Assay Kit (Thermo, USA), and 80 µg protein was loaded in each lane. Western blot analysis was performed as previously described [23]. The antibodies we used were as follows: DNMT1 (abcam, ab13537, 1:1000); DNMT3b (abcam, ab13604, 1:500); SOX2 (abcam, ab97959, 1:2000); ß-actin (Sigma-Aldrich, A5441, 1:10000).

### Colony and sphere formation assay

Colony formation assay was performed by seeding 200 cells into 6-well plates (Corning). After cultured for 14 days, plates were stained with crystal violet for 10 minutes. The number of colonies was counted by software. Sphere formation assays were conducted as seeding 2500 cells into 6-well low-adherent plates, and cultured with non-FBS medium supplemented with special growth factors. After 14 days’ incubation, the numbers of spheres were counted under microscope.

### Cell proliferation experiment

Cells were plated in 96-well plates at 400 cells per well. The assay was performed essentially as protocols of Cell Counting Kit 8 (Tongren, Shanghai, China). The absorbance of each sample using a microplate reader at a wavelength of 450 nm.

### Cell cycle analysis

Logarithmically growing cells were harvested, washed with PBS, and then fixed with 70% ethanol overnight. After staining with Propidium Iodide (PI) for 30 minutes in dark, cell cycle was analyzed by FACS.

### Sensitivity to chemotherapeutic reagents

5000 cells were plated in 96-well plate. Cisplatin was added in different concentrations (2 µg/ml, 4 µg/ml, 6 µg/ml, 8 µg/ml and 10 µg/ml), with phosphate buffered solution (PBS) as a control. A CCK8 assay was performed after 24 h culture.

### Cell migration assay

The migratory capacity of cells was analyzed using Costar chambers with 8 µm pore size (Costar322, USA). Samples each containing 2 × 10^5^ cells in 0.2 ml of serum-free medium were added to the upper compartments. The lower compartments were filled with 0.8 ml of RPMI1640 containing 20% FBS as a source of chemoattractants. The chambers were incubated for 24 hours at 37°C and 5% CO_2_. After incubation, cells on the top surface of the filters were wiped off with cotton swabs. Cells that attached to the lower surface of the filter were counted after staining with crystal violet staining.

### Xenograft assay in mice

Nude mice were kept in micro-isolator cages according to the guidelines of CAMS & PUMC, and all experiments were approved by the animal care committee of CAMS & PUMC. The freshly prepared (1 × 10^6^/each) cells were injected subcutaneously into the left axillary fossa of female mice (3–4 weeks old) in 100 μl PBS. The mice were monitored twice a week for palpable tumor formation and were killed at 4 weeks after transplantation to determine tumor formation.

### Immunohistochemistry (IHC)

Sections of formalin-fixed, paraffin-embedded tissues were deparaffinized and dehydrated. Endogenous peroxidase activity was blocked by incubating sections in 3% H_2_O_2_. After antigen retrieval and nonspecific reaction blockage, sections were incubated with antibody against SOX2 (dilution 1:1000; ab97959, abcam, UK) and DNMT1 (dilution 1:1000; ab13537, abcam, UK) at 4°C overnight. Sections were then incubated with a biotinylated secondary antibody (anti-goat immunoglobulin) at room temperature for 30 min and then with an avidin-biotin-peroxidase complex at room temperature for 30 min. A 3, 3′-diaminobenzidine-hydrogen was used as chromogen, followed by light hematoxylin counter- staining. For each sample, at least three high-power fields and 500 cells were randomly counted. The immunoreactivity rate in each sample was expressed as a percentage of all the cells counted.

### Statistical analysis

Statistical software SPSS16.0 was used in data processing and for analyzing the significance among groups. *P* < 0.05 was considered statistically significant.

## SUPPLEMENTARY MATERIALS FIGURE



## Abbreviations

CSCs: cancer stem cells
ESCC: esophageal squamous cell carcinoma
DNMT1: DNA methyltransferase 1
5-aza-dC: 5-aza-2’-deoxycytidine
SP: side population
