# Supplementary material for: DNMT1 ablation suppresses tumorigenesis by inhibiting the self-renewal of esophageal cancer stem cells
Source: Oncotarget. 2018 Jan 2;9(27):18896–907. doi: 10.18632/oncotarget.24116 (PMC5922364; doi:10.18632/oncotarget.24116)
Supplement: Supplementary file 1 [file oncotarget-09-18896-s001.pdf]

DNMT1 ablation suppresses tumorigenesis by inhibiting the self-renewal of esophageal cancer stem cells

SUPPLEMENTARY MATERIALS

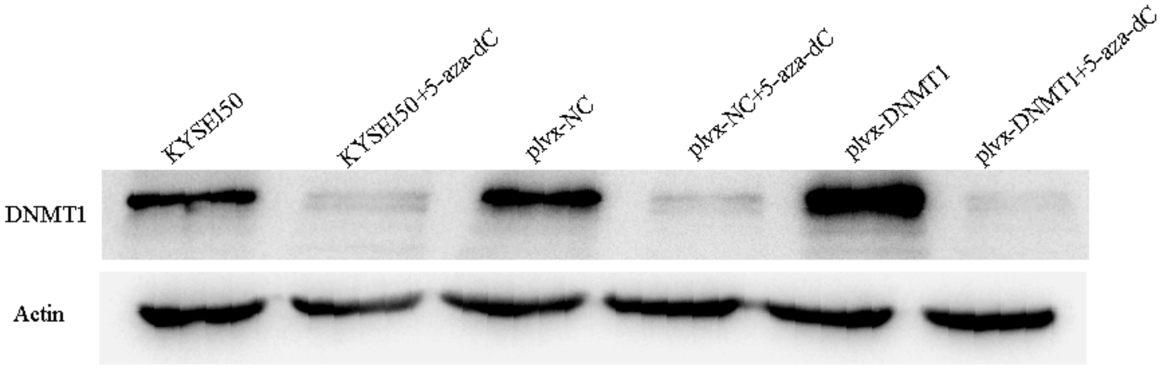

Supplementary Figure 1: Overexpressed DNMT1 could not rescue the DNMT1 degradation by 5-aza-dC. Lenti-plvx-DNMT1 virus transfection can increase the expression of DNMT1 in KYSE150 cells. After the 5-aza-dC treatment, the expression of DNMT1 was also dramatically decreased, similarly to non-transfected cells.
